# Supplementary material for: Oligomerization-dependent and synergistic regulation of Cdc42 GTPase cycling by a GEF and a GAP
Source: EMBO Rep. 2026 Feb 9;27(6):1463–77. doi: 10.1038/s44319-026-00695-7 (PMC13022455; doi:10.1038/s44319-026-00695-7)
Supplement: Supplementary file 1 — Appendix [file 44319_2026_695_MOESM1_ESM.pdf]

## Appendix

**Table of Content**


**Appendix Supplementary Text 1:** The GTPase activity model. 2
**Appendix Figure S1:** The GTPase activity model: The GTP concentration declines exponentially with time in GTPase reactions. 4
**Appendix Figure S2:** The GTPase activity model: The variation between GTPase assays is small. 5
**Appendix Figure S3:** SEC-MALS analysis of Cdc24: Cdc24 oligomerization is weak. 6
**Appendix Supplementary Text 2:** Cdc24 mutants . 7
**Appendix Figure S4:** Cdc24 mutants: Cdc24-DH3 expression yielded the full-length protein, whereas for Cdc24-DH5 only fragments could be detected. 9
**Appendix Table S1:** Cdc24 mutants: Interaction rates $k3a$ and $k3b$ of Cdc42 - Cdc24/Cdc24-DH3 mixtures (determined using Eq. 4). 10
**Appendix Supplementary Text 3:** Non-canonical effects in GTPase assays 11
**Appendix Figure S5:** Non-canonical effects in GTPase assays: In absence of an GTPase enzyme, BSA and Casein do not lead to GTP hydrolysis (or affect any downstream processing steps of the assay). 12
**Appendix Figure S6:** Non-canonical effects in GTPase assays: Inert proteins (BSA, Casein) slightly boost Cdc42's GTPase activity. 13
**Appendix Figure S7:** Non-canonical effects in GTPase assays: The inert protein Casein slightly boosts the GTPase activity of Ras. 14
**Appendix Figure S8:** Non-canonical effects in GTPase assays: The inert proteins BSA slightly enhances the effect of Cdc24, but not of Rga2($I$), on Cdc42's GTPase activity. 15
**Appendix Table S2.** Non-canonical effects in GTPase assays: GTP hydrolysis cycling rates $k1$ and $k2$ of Cdc42 and Ras. 16
**Appendix Table S3.** Non-canonical effects in GTPase assays: Interaction rates $k3,X$ of GTPase - effector protein mixtures. 16
**Appendix Table S4.** Non-canonical effects in GTPase assays: Cdc42 - effector protein $X1$ - effector protein $X2$ interaction rates $k3,X1$ , $k3,X2$ , and $k3,X1,X2$ . 16
**Appendix Supplementary Text 4:** The rate-limiting step model. 17
**Appendix Table S5.** The rate-limiting step model: Comparison of acceleration factors determined experimentally (Eq. 5) with those following the rate-limiting step model (Eq. 6, 7). 21
**Appendix Figure S9:** Amino acid sequence of the Rga2($I$) construct. 22
**Appendix Figure S10:** Regime diagram illustrating the dominant rate $K$ across varying Cdc24 and Rga2($I$) concentrations. 23
**Appendix Figure S11:** Comparison of the synergy between used GAPs and Cdc24. The C-terminal Flag-tag of Rga2($II$) might weaken Rga2-Cdc24 binding, reducing Cdc24-Rga2($II$) synergy. 24
**Appendix Figure S12:** Schematic of the plasmid construction. 25
**Appendix Table S6.** Primer overview. 26
**Appendix Supplementary Text 5:** Amino acid sequences of used proteins. 27
**Appendix Figure S13:** SDS-PAGE of used proteins 29
**Appendix References** 30

## Appendix Supplementary Text 1: The GTPase activity model

We developed a Cdc42 GTPase activity model for determining the GTPase cycling rates *k*. The model is described in detail in [Tschirpke et al., 2024]. Key aspects are summarized here:

### GTPase model

Cdc42 GTPase cycling involves three steps: (1) A GTP molecule from solution binds to Cdc42. (2) Cdc42 hydrolyses GTP. (3) Cdc42 releases GDP.

$$[\mathrm{GTP}] + [\mathrm{Cdc42}] \rightleftharpoons [\mathrm{Cdc42{-}GTP}]$$

$$[\mathrm{Cdc42{-}GTP}] \longrightarrow [\mathrm{Cdc42{-}GDP}]$$

$$[\mathrm{Cdc42{-}GDP}] \rightleftharpoons [\mathrm{Cdc42}] + [\mathrm{GDP}]$$

It can further be upregulated by effector proteins: GAPs have been shown to enhance GTP hydrolysis by Cdc42 (step 2), GEFs enhance the release of GDP from Cdc42 (step 3) [Park and Bi, 2007, Martin, 2015, Chiou et al., 2017].

To quantitatively describe the GTPase reaction cycle, we coarse-grained the GTPase reaction steps with

$$[\mathrm{GTP}] + n\,[\mathrm{Cdc42}] \longrightarrow [\mathrm{GDP}] + n\,[\mathrm{Cdc42}]$$

To account for possible Cdc42 dimerization and cooperativity, we included the following reactions into the model:

(1) We assume that Cdc42 can dimerize, as other small GTPases have been shown to dimerize [Zhang and Zheng, 1998, Zhang et al., 1999, Zhang et al., 2001, Kang et al., 2010]:

$$2\,[\mathrm{mCdc42}] \rightleftharpoons [\mathrm{dCdc42}]$$

and both monomeric and dimeric Cdc42 can contribute to the overall GTP hydrolysis with different rates:

$$[\mathrm{GTP}] + [\mathrm{mCdc42}] \xrightarrow{k_1'} [\mathrm{GDP}] + [\mathrm{mCdc42}]$$

$$[\mathrm{GTP}] + [\mathrm{dCdc42}] \xrightarrow{k_2'} [\mathrm{GDP}] + [\mathrm{dCdc42}]$$

Assuming that the majority of Cdc42 is in its monomeric form ($[\mathrm{mCdc42}] < C_d$, with $C_d$ as the concentration at which half of the total Cdc42 is dimeric), we can approximate

$$\begin{aligned} [\mathrm{dCdc42}] &= \frac{[\mathrm{mCdc42}]^2}{2C_d} \\ [\mathrm{mCdc42}] &\approx [\mathrm{Cdc42}] - \frac{[\mathrm{Cdc42}]^2}{C_d} \end{aligned} \tag{2}$$

(2) Next to cooperativity from dimerization, cooperativity can also emerge when Cdc42 proteins come in close contact with each other - they can affect each other's behavior without forming a stable homodimer, effectively functioning as an effector protein for themselves:

$$[\mathrm{GTP}] + 2\,[\mathrm{mCdc42}] \xrightarrow{k_3'} [\mathrm{GDP}] + 2\,[\mathrm{mCdc42}]$$

$$[\mathrm{GTP}] + [\mathrm{mCdc42}] + [\mathrm{dCdc42}] \xrightarrow{k_4'} [\mathrm{GDP}] + [\mathrm{mCdc42}] + [\mathrm{dCdc42}]$$

(3) Effector proteins, such as GAPs and GEFs, affect the speed of the GTP hydrolysis cycle:

$$[\mathrm{GTP}] + [\mathrm{Cdc42}][\mathrm{X}]^m \xrightarrow{k_5'} [\mathrm{GDP}] + [\mathrm{Cdc42}][\mathrm{X}]^m$$

Here X is an effector protein.

Our data shows that the amount of remaining GTP follows an exponential decline over time (Appendix Fig. S1):

$$[\mathrm{GTP}]_t = [\mathrm{GTP}]_{t_0} \exp(-Kt)\text{ , using } [\mathrm{GTP}]_{t_0} = 1 \tag{3}$$

Considering reactions (1) - (3), we can thus define $K$ in Eq. 3 as

$$K = k_1'[\mathrm{mCdc42}] + k_2'[\mathrm{dCdc42}] + k_3'[\mathrm{mCdc42}]^2 + k_4'[\mathrm{mCdc42}][\mathrm{dCdc42}] + k_5'[\mathrm{Cdc42}][\mathrm{X}]^m$$

Using Eq. 2, and considering only up to second-order terms, results in

$$
\begin{aligned}
K &= k'_1[\text{Cdc42}] + \left(\frac{k'_2}{2C_d} + k'_3 - \frac{k'_1}{C_d}\right)[\text{Cdc42}]^2 + k'_5[\text{Cdc42}][X]^m \\
&= k_1[\text{Cdc42}] + k_2[\text{Cdc42}]^2 + k_{3,X}[\text{Cdc42}][\text{X}]^m
\end{aligned}
\tag{4}
$$

where $k_1$ refers to GTP hydrolysis cycling rates of monomeric Cdc42, $k_2$ includes effects of cooperativity and dimerization and $k_3$ represents the rate of Cdc42 - effector interaction. We refer to $K$ as 'overall GTP hydrolysis rate'.

### Variability between assays

We used Eq. 4 with [X]=0 to determine the rates of Cdc42 alone. We then conducted assays with Cdc42 and an effector protein to determine $k_3$. While doing so we needed to account for assay variability, i.e. for the observation that the rates for Cdc42 can vary between assays. Possible reasons for this include small concentration differences introduced though pipetting of small volumes (as are required for this assay), temperature and shaker speed fluctuations during the incubation step, and/or intrinsic changes in the protein activities due to other external conditions. To account for this variance, we introduced the parameter $c_{corr}$. It maps all factors that lead to variations between assays onto the Cdc42 concentration.

The assay data, including samples containing only Cdc42 and Cdc42 - (effector) protein mixtures, was fitted with

$$K = k_1 c_{corr}[\text{Cdc42}] + k_2(c_{corr}([\text{Cdc42}])^2 + k_{3,X} c_{corr}[\text{Cdc42}][\text{X}]^m$$

to determine $c_{corr}$ and $k_{3,X}$ (using $k_1$ and $k_2$ determined earlier).

Only assays with $c_{corr}$ values from 0.5 to 1.5 were used for analysis. Generally, with $c_{corr}$ values were distributed around 1.0, confirming that the variation between assays is small (Appendix Fig. S2).

### Pooled estimates

The pooled estimates of rates $k_1$, $k_2$, and $k_3$ were determined through weighting their standard error, as described in the following:

Within an assay, the rate parameters per run are calculated, but also a weighted average can be taken from these values to create a pooled estimate. Concretely:

For pooling, we model the $n$ parameter estimates $y_i$ to originate from a single pooled estimate $a$ as:

$$\begin{bmatrix} y_1 \\ y_2 \\ \vdots \\ y_n \end{bmatrix} = a \begin{bmatrix} 1 \\ 1 \\ \vdots \\ 1 \end{bmatrix} + \begin{bmatrix} \varepsilon_1 \\ \varepsilon_2 \\ \vdots \\ \varepsilon_n \end{bmatrix}$$

with $\epsilon_i \sim N(0, \sigma_i)$ where $\sigma_i$ is the standard error of the parameter estimate $y_i$. As uncertain estimates should be weighted less, the natural weights $w_i$ to each $y_i$ should be $1/\sigma_i$, after which all weighted errors follow a standard normal distribution:

$$\begin{bmatrix} w_1 y_1 \\ w_2 y_2 \\ \vdots \\ w_n y_n \end{bmatrix} = a \begin{bmatrix} w_1 \\ w_2 \\ \vdots \\ w_n \end{bmatrix} + \begin{bmatrix} w_1 \varepsilon_1 \\ w_2 \varepsilon_2 \\ \vdots \\ w_n \varepsilon_n \end{bmatrix} \Longrightarrow \begin{bmatrix} y_1^* \\ y_2^* \\ \vdots \\ y_n^* \end{bmatrix} = a \begin{bmatrix} w_1 \\ w_2 \\ \vdots \\ w_n \end{bmatrix} + \begin{bmatrix} \varepsilon_1^* \\ \varepsilon_2^* \\ \vdots \\ \varepsilon_n^* \end{bmatrix} \Longrightarrow \vec{y}^* = a\vec{w} + \vec{\varepsilon}^*$$

Getting the estimate for $a$, namely $\hat{a}$, is the result of a simple regression (i.e. weighted least squares), minimizing the sum of squared errors (see e.g. [Heij et al., 2004]):

$$\hat{a} = \frac{\sum_{i=1}^{n} w_i y_i^*}{\sum_{i=1}^{n} w_i^2} = \frac{\sum_{i=1}^{n} w_i^2 y_i}{\sum_{i=1}^{n} w_i^2} = \frac{\sum_{i=1}^{n} \frac{y_i}{\sigma_i^2}}{\sum_{i=1}^{n} \frac{1}{\sigma_i^2}}$$

$$\sigma_{\hat{a}} = \frac{\frac{1}{n-1} \sum_{i=1}^{n} (w_i y_i - w_i \hat{a})^2}{\left(\sum_{i=1}^{n} w_i^2\right)^2}$$

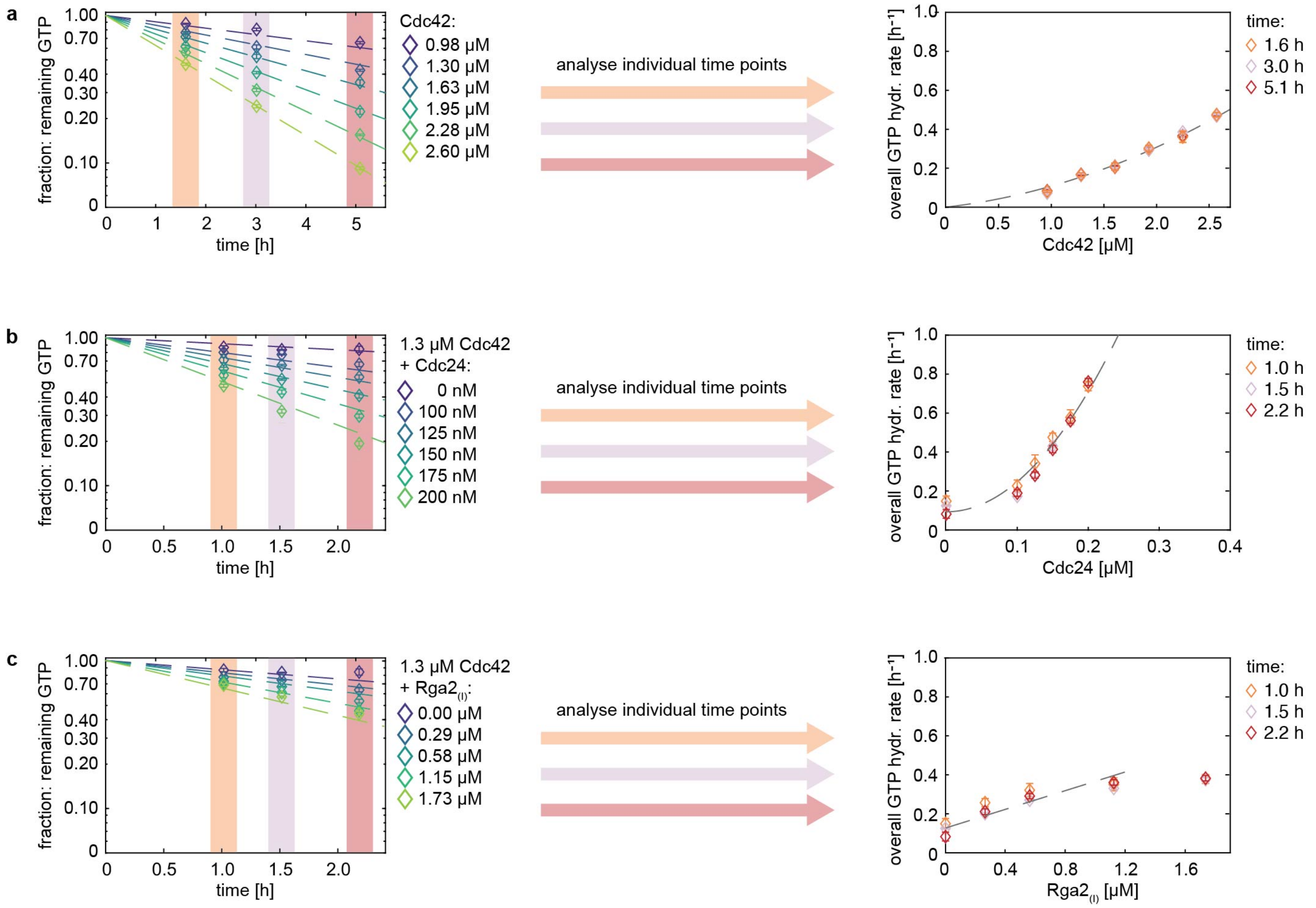


**Appendix Figure S 1. The GTPase activity model: The GTP concentration declines exponentially with time in GTPase reactions.** Amount of remaining GTP for (a) Cdc42 concentrations (b) Cdc42 - Cdc24 mixtures, and (c) Cdc42 - Rga2$_{(I)}$ mixtures, each for three time points (measured as one individual assay per time point). The remaining GTP content declines exponentially with time (left). Data of each individual time point shows the same overall GTP hydrolysis cycling rate for each GTPase - effector mixture. Thus, only one time point per assay condition is needed, to fit the data (right).

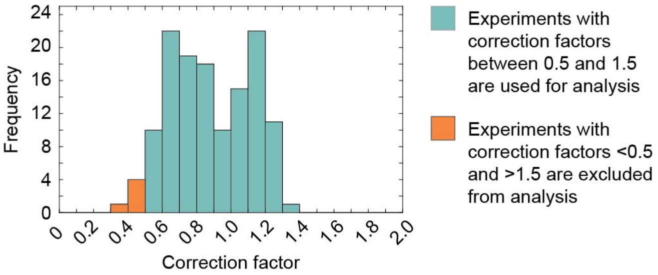


**Appendix Figure S 2. The GTPase activity model: The variation between GTPase assays is small.** $c_{corr}$ values are distributed around 1.0.

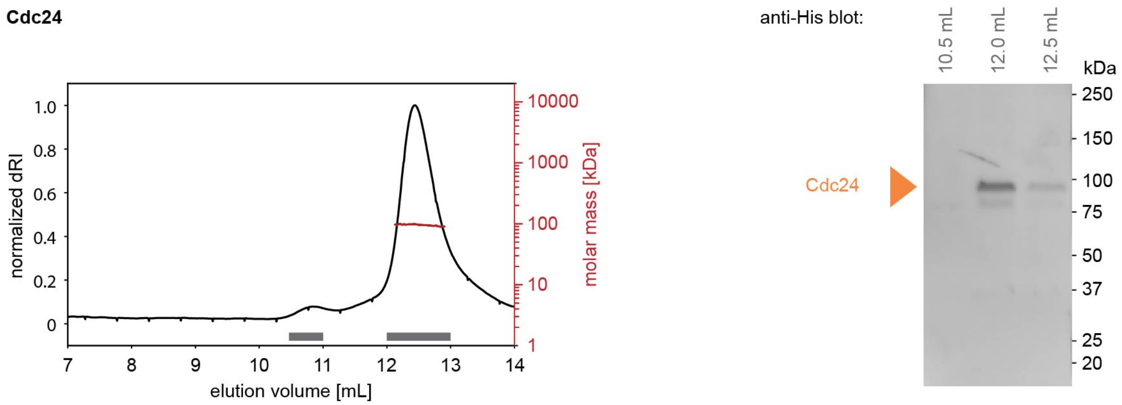


**Appendix Figure S 3. SEC-MALS analysis of Cdc24: Cdc24 oligomerization is weak.** Size-exclusion profile and MALS analysis (left) and Western blot analysis of SEC-MALS elution fractions (right).

## Appendix Supplementary Text 2: Cdc24 mutants

We found that Cdc24's effect of the overall GTP hydrolysis rate $K$ increases non-linearly with its concentration (Fig. 3b), which we speculate to be linked to Cdc24 di- or oligomerization: Cdc24 has the capability to oligomerize via its DH domain [Mionnet et al., 2008]. Dimers and oligomers could have an increased GEF activity through releasing Cdc24 from its self-inhibited state [Shimada et al., 2004].
This hypothesis is in contrast with *in vitro* work on peptides based on Cdc24 fragments, showing that these peptides exhibit an oligomerization-independent GEF activity [Mionnet et al., 2008]. These findings would exclude that Cdc24 oligomers exhibit an increased GEF activity.
However, Mionnet *et al.* used peptides that were based on some of Cdc24's domains (DH and PH domain, aa 285-681), and not full-length protein, for their *in vitro* GEF activity assays. Other domains that are not directly involved in oligomerization or GEF function can still affect these properties. For example, the PB1 domain was suggested to reduce Cdc24 GEF activity in a self-inhibitory fashion [Shimada et al., 2004]. Next, oligomerization was induced through a chemical; the peptides contained an additional oligomerization domain (FKBP) that was not related to Cdc24 and could be triggered to oligomerize through the addition of a chemical (synthetic oligomerization inducer AP20187). Thus, the findings on Cdc24 peptides might not fully translate to full-length Cdc24.

To investigate our hypothesis of an oligomerization-induced increase of Cdc24's GEF activity, we investigated two Cdc24 mutants: **Cdc24-DH3** (mutations L339A and E340A) with a reported 2.5× reduction in oligomerization and **Cdc24-DH5** (mutation F322A) with a reported 10× reduction in oligomerization [Mionnet et al., 2008].
For clarity, we like to note that the oligomerization studies conducted by Mionnet *et al.* utilized full-length Cdc24, while the *in vitro* GEF activity assays utilized peptides based on Cdc24 fragments and chemically induced oligomerization. While we question the generality of an oligomerization-independent GEF activity of Cdc24 (obtained using Cdc24 peptides and chemically induced oligomerization), we do not question their findings on Cdc24 oligomerization (obtained using full-length protein).

We recombinantly expressed Cdc24-DH3 and Cdc24-DH5 and purified them using His-affinity chromatography. Cdc24-DH3 expressed as the full-length protein, while for Cdc24-DH5 only fragments remained (Appendix Fig. S4). Mionnet *et al.* had already observed that *S. cerevisiae* cells expressing Cdc24-DH5 as sole copy were not viable and suggested that this was due to Cdc24-DH5's inability to localize correctly [Mionnet et al., 2008]. Although our data does not disprove this conclusion, it presents an additional possibility: If the DH5 mutation disrupts Cdc24 folding and expression, cells expressing Cdc24-DH5 might not be viable because they lack full-length Cdc24.

We continued our investigation utilizing Cdc24-DH3 (which was further purified using size exclusion chromatography, Appendix Fig. S13): We conducted GTPase assays to determine the concentration-dependent increase of the overall GTP hydrolysis rate $K$ through Cdc24-DH3 (Fig. 3c), fitting the data with our standard exponential model (Fig. 3d). Through comparing rates $k_3$, we found that Cdc24-DH3 has a 17× reduced GEF activity compared to wildtype Cdc24 (Fig. 3e, Tab. 2). Cdc24-DH3 contains mutations L339A and E340A, which are located in the Dbl homology (DH) domain. The DH domain is linked to GTPase interaction and activation, and responsible for Cdc24 oligomerization. It is thus possible that (1) the mutations lead to both a reduction in oligomerization and a reduced GEF activity, or (2) the mutations lead to reduced oligomerization, resulting in a reduced GEF activity. However, given that peptides comprising Cdc24's DH domain did not show a reduced GEF activity when the DH3 mutations were introduced [Mionnet et al., 2008], we believe that the reduced GEF activity of full length Cdc24-DH3 stems from its reduced ability to oligomerize.

We hypothesized that the non-linear rate increase through Cdc24 stems from Cdc24 oligomerization: Cdc24 oligomers could have an increased GEF activity through being released of the self-inhibited state Cdc24 monomers are in [Shimada et al., 2004]. With increasing Cdc24 concentration the amount of Cdc24 oligomers increases, resulting in a non-linear increase of the overall GTP hydrolysis rate $K$. Cdc24-DH3 has a 2.5× reduction in its oligomerization capacity [Mionnet et al., 2008]. If the non-linear rate increase through Cdc24 is indeed linked to its oligomerization, Cdc24-DH3 should show a more linear (/less

quadratic) rate increase. We therefore fitted data of both Cdc42 - Cdc24 and Cdc42 - Cdc24-DH3 mixtures, only using assays with at least five data points, with a modified version of our model:

$$
\begin{gathered}
[\text{GTP}]_t = [\text{GTP}]_{0h} \exp(-Kt) \\
\text{using } [GTP]_{0h} = 1 \\
\text{and } K = K_1 + K_2 + K_{3a} + K_{3b} = k_1[\text{Cdc42}] + k_2[\text{Cdc42}]^2 + k_{3a}[\text{Cdc42}][\text{X}] + k_{3b}[\text{Cdc42}][\text{X}]^2
\end{gathered} \tag{5}
$$

Here $X$ is either Cdc24 or Cdc24-DH3. $K_{3a}$ scales linearly with the Cdc24/Cdc24-DH3 concentration, and $K_{3b}$ scales quadratically with the Cdc24/Cdc24-DH3 concentration. We determined the ratio of the two rates ($\frac{k_{3b}}{k_{3a}}$, Appendix Tab. S1). The larger this ratio, the more quadratic (less linear) the overall GTP hydrolysis rate $K$ increases with Cdc24/Cdc24-DH3 concentration. Cdc24-DH3 data exhibits $\frac{k_{3b}}{k_{3a}} = 10$ and Cdc24 data exhibits $\frac{k_{3b}}{k_{3a}} = 17$, suggesting that the rate increase through Cdc24-DH3 is indeed more linear than the rate increase through wildtype Cdc24. These findings support our hypothesis that the non-linear rate increase of the overall GTP hydrolysis rate $K$ through Cdc24 might originate from Cdc24 oligomerization.

Taken together, our data suggests that

1. **Cdc24's GEF activity might be oligomerization-dependent.** Our data on the Cdc24-DH3 mutant, which has a 2.5× reduced oligomerization capacity [Mionnet et al., 2008], shows that: (1) Cdc24-DH3 has a reduced GEF activity. (2) The overall GTP hydrolysis rate $K$ increases more linearly (/less quadratically) with Cdc24-DH3 concentration than with the concentration of wildtype Cdc24. Both findings support our hypothesis that Cdc24's GEF activity is oligomerization-dependent.
2. **Findings obtained using peptides of Cdc24 fragments do not translate to full-length Cdc24.** Mionnet *et al.* found that a chemically induced oligomerization of peptides based on Cdc24 fragments does not affect their GEF activity [Mionnet et al., 2008]. We found that a Cdc24 mutant with reduced oligomerization capacity has a reduced GEF activity, questioning the generalizability of data based on protein fragments.

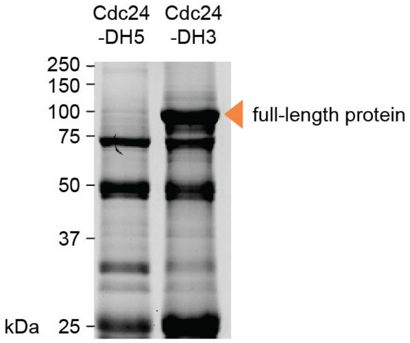


**Appendix Figure S 4. Cdc24 mutants: Cdc24-DH3 expression yielded the full-length protein, whereas for Cdc24-DH5 only fragments could be detected.** SDS-PAGE of Cdc24 mutants Cdc24-DH5 and Cdc24-DH3 after His-affinity chromatography.

**Appendix Table S 1. Cdc24 mutants:** Interaction rates $k_{3a}$ and $k_{3b}$ of Cdc42 - Cdc24/Cdc24-DH3 mixtures (determined using Eq. 5).

| | $k_{3a}$ [$\mu M^{-3}$ h$^{-1}$] | $k_{3a}$ std. err. [$\mu M^{-3}$ h$^{-1}$] | $k_{3b}$ [$\mu M^{-4}$ h$^{-1}$] | $k_{3b}$ std. err. [$\mu M^{-4}$ h$^{-1}$] | $k_{3b}/k_{3a}$ [$\mu M^{-1}$ h$^{-1}$] |
|---|---|---|---|---|---|
| Cdc24: pooled estimate (n=16) | 0.409 | 0.049 | 6.768 | 0.191 | 17 |
| Cdc24-DH3: pooled estimate (n=4) | 0.069 | 0.014 | 0.663 | 0.083 | 10 |

## Appendix Supplementary Text 3: Non-canonical effects in GTPase-Glo assays

To ensure the effects we observed in our assays are protein-specific, we conducted assays with bovine serum albumin (BSA) and Casein - two proteins considered inert. These proteins are expected to not affect the assay or interact with GTPases.

In absence of GTPases both BSA and Casein did not lead to hydrolyzed GTP, showing that they do not cause GTP hydrolysis themselves and that they do not affect any downstream processes of the assay (Appendix Fig. 5). They slightly increase the overall GTP hydrolysis rate of Cdc42 (Appendix Fig. 6) and to a smaller extend that of Ras (Appendix Fig. S7). The BSA and Casein concentrations used here (0-5 $\mu$M) are far below concentrations where crowding effects are expected [Chebotareva et al., 2004], excluding crowding effects. We suspect that through sticking to reaction chamber walls BSA and Casein increase the effective GTPase concentration in the assay, thus causing a slight increase in the overall GTP hydrolysis rate. Supporting this line of reasoning is the observation that the effect of Casein on Cdc42 is larger than its effect on Ras: Cdc42 has a higher GTPase activity than Ras (Appendix Tab. S2). An effective increase in Cdc42's effective concentration will thus have a larger effect on it's overall GTP hydrolysis rate $K$ than a similar concentration increase of Ras. The effect of BSA and Casein is at least 3.7× smaller than the effect of Rga2$_{(I)}$ (the weakest of Cdc42's effectors) (Appendix Tab. S3), suggesting that their non-canonical effect on the assay does not play a major role.

We conducted some preliminary assays with (a) Cdc42 - Cdc24 - BSA and (b) Cdc42 - Rga2$_{(I)}$ - BSA mixtures (Appendix Fig. S8): In our model the effectors contribute to the overall GTP hydrolysis rate $K$ of Cdc42 through three terms: $K_{3,X1}$, $K_{3,X2}$, and $K_{3,X1,X2}$ where $X1$ and $X2$ are (a) Cdc24 and BSA and (b) Rga2$_{(I)}$ and BSA. $K_{3,Cdc24}$, $K_{3,Rga2}$, and $K_{3,BSA}$ represent the rate contribution of Cdc24, Rga2$_{(I)}$, and BSA alone. They are in the three-protein mixture (Cdc42 - Cdc24 - BSA, Cdc42 - Rga2$_{(I)}$ - BSA) roughly the same as when Cdc42 was incubated with one effector alone (Cdc42 + Cdc24, Cdc42 + Rga2, or Cdc42 + BSA) (Appendix Fig. S8c). The interaction term $K_{3,Cdc24,BSA}$ of Cdc24 and BSA was roughly the same as the individual contribution of Cdc24 ($K_{3,Cdc24,BSA} \approx K_{3,Cdc24}$) and Rga2 and BSA showed no interaction ($K_{3,Rga2,BSA} \approx 0$)[1] (Appendix Tab. S4). If BSA is sticking to reaction chamber walls, it can increase the effective concentration of Cdc42 and Cdc24/Rga2 in the assay. Given that Cdc24 had a strong and Rga2 had a weak effect on Cdc42, an increase in the effective Cdc24 concentration results in a significant increase of the overall GTP hydrolysis rate (leading to a positive $K_{3,Cdc24,BSA}$), whereas an increase in the effective Rga2 concentration has almost no observable effect ($K_{3,Rga2,BSA} \approx 0$). Importantly, $K_{3,Cdc24,BSA}$ is seven times smaller than $K_{3,Cdc24,Rga2}$ (the interaction term between Cdc24 and Rga2$_{(I)}$ in Cdc42 - Cdc24 - Rga2$_{(I)}$ assays, Fig. 5), confirming that the observed synergy between Cdc24 and Rga2$_{(I)}$ is not due to non-canonical effects.

[1]The fit quality for the Cdc42-Rga2-BSA data was significantly lower than for all other data sets ($R^2 \approx 0.5$). Hence, we remain cautious drawing conclusions from this data.

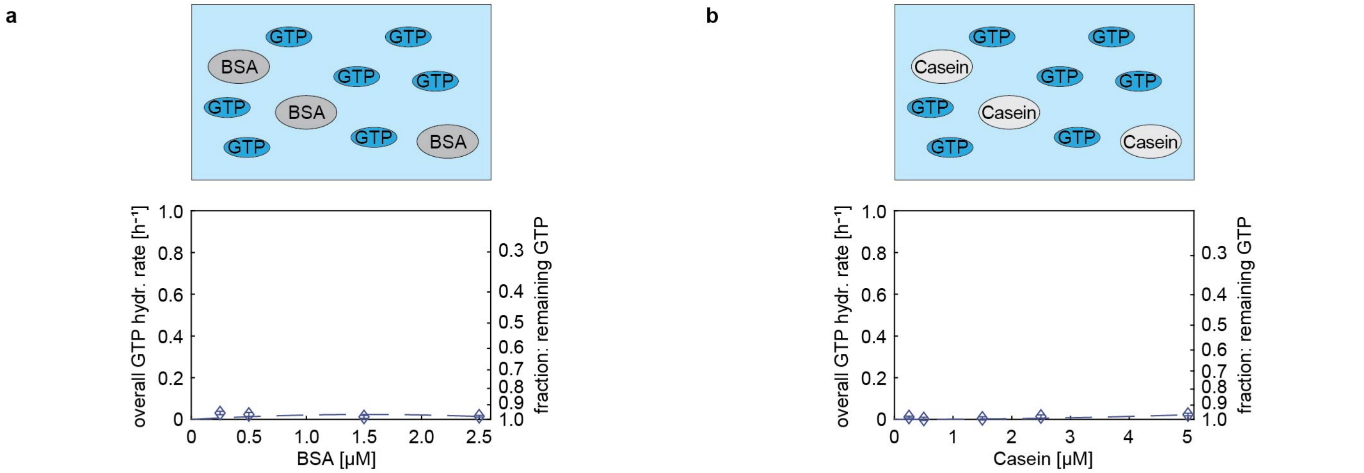


**Appendix Figure S 5. Non-canonical effects in GTPase assays: In absence of an GTPase enzyme, BSA and Casein do not lead to GTP hydrolysis (or affect any downstream processing steps of the assay).** (a) BSA incubated with GTP. (b) Casein incubated with GTP.

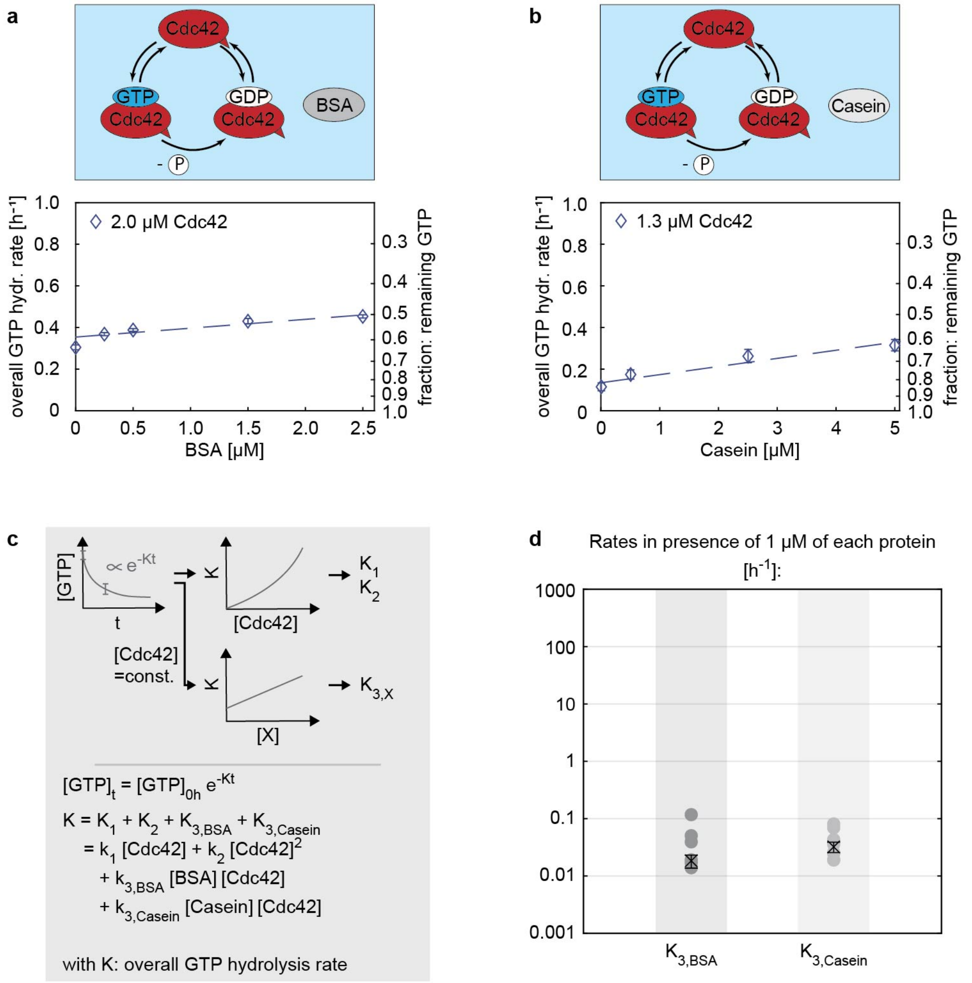


**Appendix Figure S 6. Non-canonical effects in GTPase assays: inert proteins (BSA, Casein) slightly boost Cdc42's GTPase activity.** (a,b) The overall GTP hydrolysis rate $K$ of Cdc42 in presence of varying BSA (a) and Casein (b) concentrations. The increase in Cdc42's overall GTP hydrolysis rate could be due to (1) unknown non-specific interactions or (2) because BSA and Casein coat to the reaction chamber walls, preventing Cdc42 from binding, thus increasing the active Cdc42 concentration in the reaction chamber. (c) Illustration of the data processing and fitting model. (d) Summary of the rates $K$. The values shown refer to the rate values in presence of 1 $\mu$M of each protein, e.g. '$K_{3,BSA}$' refers to '$k_{3,BSA}$[BSA][Cdc42]' with [Cdc42]=[BSA]=1 $\mu$M. Crosses with error bars represent the weighted mean and the standard error of the mean (Appendix  Supplementary Text 1), filled circles show individual measurements.

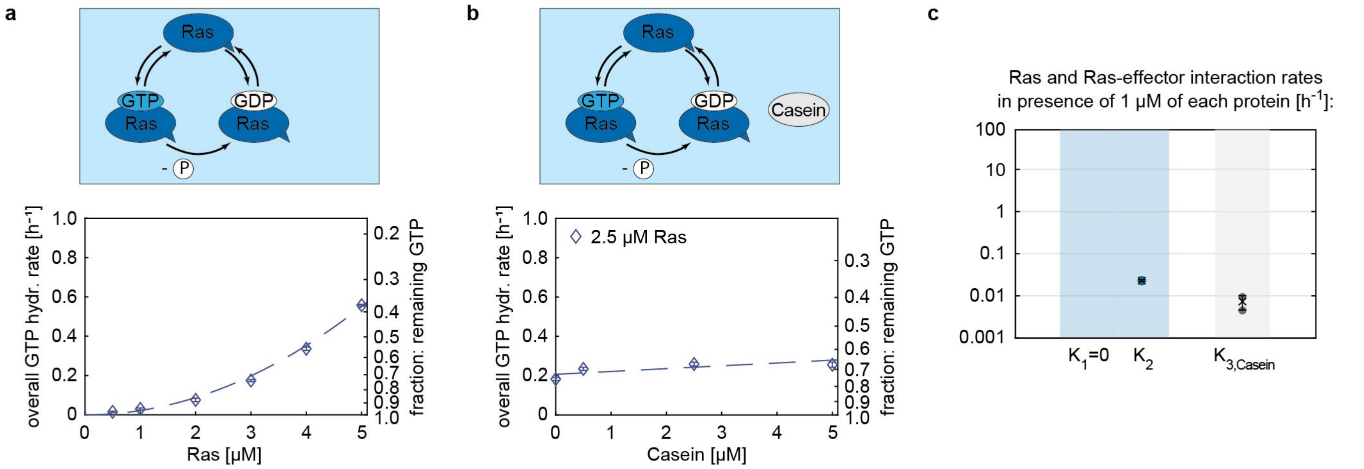


**Appendix Figure S 7. Non-canonical effects in GTPase assays: the inert protein Casein slightly boosts the GTPase activity of Ras.** (a) The overall GTP hydrolysis rate ($K$) of Ras. (b) The overall GTP hydrolysis rate ($K$) of Ras in presence of varying Casein concentrations. The increase in the overall GTP hydrolysis rate could be due to (1) unknown non-specific interactions or (2) because Casein coats to the reaction chamber walls, preventing Ras from binding, thus increasing the active Ras concentration in the reaction chamber. (c) Summary of the rates $K$. The values shown refer to the rate values in presence of 1 µM of each protein, e.g. '$K_{3,Casein}$' refers to '$k_{3,Casein}$[Casein][Ras]' with [Ras]=[Casein]=1 µM. Crosses with error bars represent the weighted mean and the standard error of the mean (Appendix Supplementary Text 1), filled circles show individual measurements.

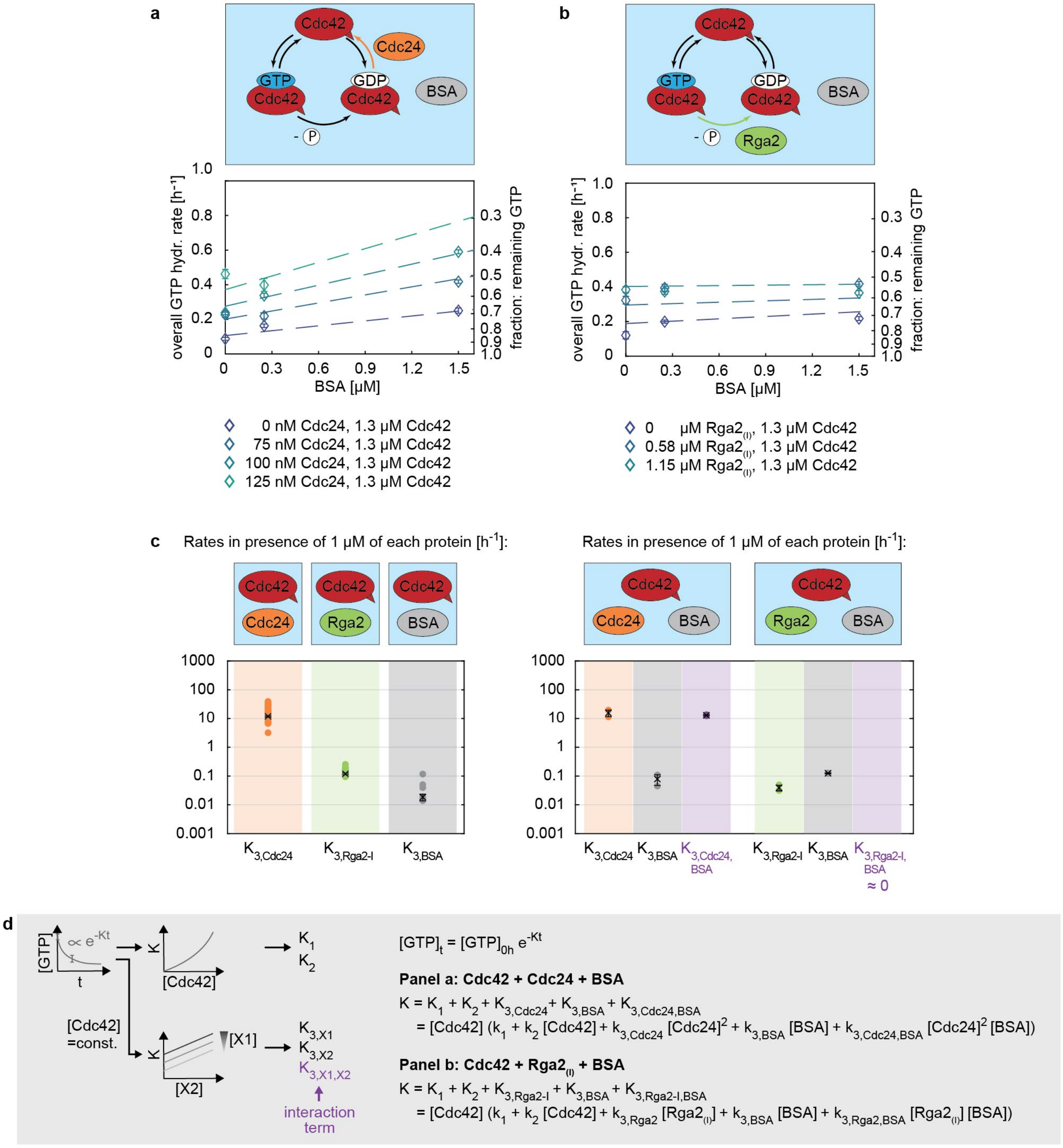


**Appendix Figure S 8. Non-canonical effects in GTPase assays: the inert proteins BSA slightly enhances the effect of Cdc24, but not of Rga2$_{(I)}$, on Cdc42's GTPase activity.** (a,b) Increase in the overall GTP hydrolysis cycling rate $K$ of Cdc42 in presence of (a) Cdc24 and BSA, and (b) Rga2 and BSA. (c) Summary of the rates $K$ obtained in the three-protein assay (right) in comparison to those of the two-protein assay (left): In three-protein assays the rate contribution of the individual proteins is comparable to those obtained in the two-protein assay. Additionally, an interaction rate is obtained (shown in purple). In the case of Cdc42-Cdc24-BSA and Cdc42-Rga2-BSA mixtures, this interaction rate is comparable to the Cdc24 contribution/zero, indicating a weak/ almost no synergy. This effect could be due to non-specific protein-protein binding or because BSA coats to the reaction chamber walls, preventing the other proteins from binding, thus increasing their active concentration in the reaction chamber. The values shown refer to the rate values in presence of 1 μM of each protein, e.g. '$K_{3,BSA}$' refers to '$k_{3,BSA}$[BSA][Cdc42]' with [Cdc42]=[BSA]=1 μM. Crosses with error bars represent the weighted mean and the standard error of the mean (Appendix Supplementary Text 1), filled circles show individual measurements. (d) Illustration of the data processing and fitting model.

**Appendix Table S 2. Non-canonical effects in GTPase assays:** GTP hydrolysis cycling rates $k_1$ and $k_2$ of Cdc42 and Ras.

| | $k_1$ [μM$^{-1}$ h$^{-1}$] | $k_1$ std. err. [μM$^{-1}$ h$^{-1}$] | $k_2$ [μM$^{-2}$ h$^{-1}$] | $k_2$ std. err. [μM$^{-2}$ h$^{-1}$] |
|---|---|---|---|---|
| Ras, pooled estimate (n=2) | 0.000 | 0.000 | 0.021 | 0.001 |
| Cdc42, pooled estimate (n=5) | 0.110 | 0.002 | 0.013 | 0.001 |

**Appendix Table S 3. Non-canonical effects in GTPase assays:** Interaction rates $k_{3,X}$ of GTPase - effector protein mixtures.

| | Effector protein X | $k_{3,X}$ [μM$^{-2}$ h$^{-1}$] | $k_{3,X}$ std. err. |
|---|---|---|---|
| **Cdc42** | | | |
| pooled est. (n=6) | BSA | 0.018 | 0.005 |
| pooled est. (n=8) | Casein | 0.032 | 0.007 |
| **Ras** | | | |
| pooled est. (n=2) | Casein | 0.007 | 0.002 |

**Appendix Table S 4. Non-canonical effects in GTPase assays:** Cdc42 - effector protein $X_1$ - effector protein $X_2$ interaction rates $k_{3,X_1}$, $k_{3,X_2}$, and $k_{3,X_1,X_2}$.
*: unit in case of $X_1$=Cdc24: [μM$^{-3}$ h$^{-1}$].
**: unit in case of $X_1$=Cdc24: [μM$^{-4}$ h$^{-1}$].

| | Effector protein $X_1$ | Effector protein $X_2$ | $k_{3,X_1}$ [μM$^{-2}$ h$^{-1}$] * | $k_{3,X_1}$ std. err. | $k_{3,X_2}$ [μM$^{-2}$ h$^{-1}$] | $k_{3,X_2}$ std. err. | $k_{3,X_1,X_2}$ [μM$^{-3}$ h$^{-1}$] ** | $k_{3,X_1,X_2}$ std. err. |
|---|---|---|---|---|---|---|---|---|
| pooled est. (n=7) | Cdc24 | Rga2$_{(I)}$ | 15.126 | 0.736 | 0.131 | 0.015 | 86.961 | 6.055 |
| pooled est. (n=2) | Cdc24 | BSA | 15.642 | 4.054 | 0.079 | 0.484 | 12.887 | 0.538 |
| pooled est. (n=2) | Rga2$_{(I)}$ | BSA | 0.125 | 0.166 | 0.039 | 0.009 | -0.024 | 0.003 |

## Appendix Supplementary Text 3: The rate-limiting step model

Fitting GTPase assays data where both the GEF Cdc24 and the GAP Rga2 where added resulted in a positive synergy term (Fig. 5), which could originate from two sources: (1) Cdc24-Rga2 synergy due to rate-limiting effects of the GTPase cycle, and (2) Cdc24-Rga2 synergy due to protein-protein interactions.
In the following we describe the rate-limiting step model and then discuss to which extend the rate-limiting step model explains our data and thus the positive synergy term.

### The rate-limiting step model

In the rate-limiting step model, we assume that we have a GTPase cycle in which at least one of the three GTPase cycle steps is rate-limiting: (A) GTP binding, (B) GTP hydrolysis, and (C) GDP release. We assume that the addition of a GEF accelerates only GDP release (step C), and that the addition of a GAP only accelerates GTP hydrolysis (step B).
Biochemically, all three steps in the GTPase cycle are expected to be relevant. However, here we will consider only the final two steps, as sensitivity to rate limitation by GAP/GEF is maximized when time spent in the GAP/GEF-independent step in the cycle (step A: GTP binding) is negligible (i.e. never rate-limiting).
The rate-limiting step model thus consists of two steps:

(1) a nucleotide exchange step (step C+A) which is dominated by GDP release (step C) and assumed to be accelerated exclusively by the GEF, and
(2) a GTP hydrolysis step (step B) exclusively enhanced by the GAP.

In the rate-limiting step model, addition of the GEF to the GTPase would accelerate only step (1), leaving a slow GTP hydrolysis step (step 2) that limits the overall cycling rate. Analogously, addition of GAP would accelerate only step (2), leaving a nucleotide exchange step (step 1) that limits the overall cycling speed. Addition of both GEF and GAP would speed up both steps, and therefore synergistically accelerate the overall cycling rate. The synergy is then a property of the GTPase cycle, and not due to proteins enhancing each other's activity.
Specifically, GEF-GAP synergy can appear if one of the two conditions applies:

1. the addition of a GEF speeds up the GDP release step (step 1) so much that the release step stops (or almost stops) being the rate-limiting step, or
2. the addition of a GAP speeds up the GTP hydrolysis step (step 2) so much that the hydrolysis step stops (or almost stops) being the rate-limiting step.

In these conditions, the acceleration of the GTPase cycle, accomplished by adding only a GEF or adding only a GAP, is interdependent. Therefore, we **consider the possible acceleration of the GTPase cycle by GAP and GEF individually** (i.e. assays comprising of Cdc42 + GEF and assays comprising of Cdc42 + GAP), and compare these to our observations to determine whether the rate-limiting step model can fully explain our data.
The GTPase cycle time $T_c$ (with the rate $r_c$) is thus composed of hydrolysis time $T_h$ and nucleotide exchange time $T_e$, and the respective rates $r_h$ and $r_e$, are connected through:

$$\frac{1}{r_c} = \frac{1}{r_h} + \frac{1}{r_e}$$

If we compare the ratio of the rates with one effector (GEF or GAP) added in the assay (index 1) with the basal rate without an effector added (i.e. Cdc42 only) (index 0), we obtain the cycle acceleration factor $a$:

$$a = \frac{r_{c,1}}{r_{c,0}} = \frac{\frac{1}{r_{h,0}} + \frac{1}{r_{e,0}}}{\frac{1}{r_{h,1}} + \frac{1}{r_{e,1}}} = \frac{r_{e,0} + r_{h,0}}{\frac{r_{e,0}r_{h,0}}{r_{h,1}} + \frac{r_{e,0}r_{h,0}}{r_{e,1}}} \tag{6}$$

Using

- $r_{c,0}$: the basal GTPase cycle rate (of only Cdc42)
- $r_{c,1}$: the GTPase cycle rate with an effector added (GEF or GAP)
- $r_{h,0}$: the basal GTP hydrolysis rate (of only Cdc42)

- $r_{h,1}$: the GTP hydrolysis rate with an effector added (GEF or GAP)
- $r_{e,0}$: the basal nucleotide exchange rate (of only Cdc42)
- $r_{e,1}$: the nucleotide exchange rate with an effector added (GEF or GAP)

There is an interdependence between how much the GAP and the GEF can accelerate the GTPase cycle, if the GAP and GEF are assumed to only accelerate GTP hydrolysis and nucleotide exchange respectively. E.g., how much the total GTPase cycle rate $r_c$ is accelerated by an increase in the GTP hydrolysis rate $r_h$ depends on and can be limited by the current nucleotide exchange rate $r_e$.
When we **only add a GEF** and the GEF accelerates only the nucleotide exchange rate $r_{e,1}$ (and not the GTP hydrolysis rate $r_{h,1}$, meaning $r_{h,1} = r_{h,0}$), then the maximal total GTPase cycle rate acceleration $a_{GEF}$ that the GEF can accomplish is when

$$r_{e,1} \gg r_{h,0}, r_{e,0}$$

I.e. when the nucleotide exchange rate in presence of a GEF $r_{h,1}$ is much bigger than the basal hydrolysis rate $r_{h,0}$ and much bigger than the basal nucleotide exchange rate $r_{e,0}$:

$$a_{GEF} = \frac{r_{c,1}}{r_{c,0}} = \frac{\frac{1}{r_{h,0}} + \frac{1}{r_{e,0}}}{\frac{1}{r_{h,1}} + \frac{1}{r_{e,1}}} = \frac{r_{e,0} + r_{h,0}}{\frac{r_{e,0}r_{h,0}}{r_{h,0}} + \frac{r_{e,0}r_{h,0}}{r_{e,1}}} = \frac{r_{e,0} + r_{h,0}}{r_{e,0} + \frac{r_{e,0}r_{h,0}}{r_{e,1}}} \approx \frac{r_{e,0} + r_{h,0}}{r_{e,0}} = 1 + \frac{r_{h,0}}{r_{e,0}}$$

We thus assume the GEF accelerates the cycle so much that the nucleotide exchange step is much faster than the hydrolysis step, at which point the effect of adding more GEF would saturate.
Analogously, when we **only add a GAP** and the GAP accelerates only the GTP hydrolysis rate $r_{h,1}$ (and not the nucleotide exchange rate $r_{e,1}$, meaning $r_{e,1} = r_{e,0}$), then the maximal total GTPase cycle rate acceleration $a_{GAP}$ that the GAP can accomplish is when

$$r_{h,1} \gg r_{h,0}, r_{e,0}$$

i.e. when the hydrolysis rate in presence of a GAP $r_{h,1}$ is much bigger than the basal hydrolysis rate $r_{h,0}$ and much bigger than the basal nucleotide exchange rate $r_{e,0}$:

$$a_{GAP} = \frac{r_{c,1}}{r_{c,0}} = \frac{\frac{1}{r_{h,0}} + \frac{1}{r_{e,0}}}{\frac{1}{r_{h,1}} + \frac{1}{r_{e,1}}} = \frac{r_{e,0} + r_{h,0}}{\frac{r_{e,0}r_{h,0}}{r_{h,1}} + \frac{r_{e,0}r_{h,0}}{r_{e,0}}} = \frac{r_{e,0} + r_{h,0}}{\frac{r_{e,0}r_{h,0}}{r_{h,1}} + r_{h,0}} \approx \frac{r_{e,0} + r_{h,0}}{r_{h,0}} = 1 + \frac{r_{e,0}}{r_{h,0}}$$

We thus assume the GAP accelerates the cycle so much that the hydrolysis step is much faster than the nucleotide exchange step, at which point the effect of adding more GAP would saturate.
The maximum gain in rates for GAP-only and GEF-only assays is limited by the same basal GTP hydrolysis rate $r_{h,0}$ and basal nucleotide exchange rate $r_{e,0}$, leading to the following interdependence:

$$a_{GEF} = 1 + \frac{1}{a_{GAP} - 1} = \frac{a_{GAP}}{a_{GAP} - 1} \tag{7}$$

Analogously,

$$a_{GAP} = 1 + \frac{1}{a_{GEF} - 1} = \frac{a_{GEF}}{a_{GEF} - 1} \tag{8}$$

### Can the rate-limiting step model fully explain the experimental data? I.e. could rate-limiting steps be the *only* source of the positive $K_{3,Cdc24,Rga2}$?

If the rate-limiting step model *fully* explains the synergy term observed in Cdc42 + GEF + GAP assays (Fig. 5), at least one of the two conditions, as already outlined in the section above, must be true:

1. the addition of a GEF speeds up the GDP release step (step 1) so much that the release step stops (or almost stops) being the rate-limiting step, or
2. the addition of a GAP speeds up the GTP hydrolysis step (step 2) so much that the hydrolysis step stops (or almost stops) being the rate-limiting step.

This ultimately means that the rate-limiting step model describes our data fully if at least one of the two the acceleration factors determined using our experimental data ($a_{GEF}$ or $a_{GAP}$) is consistent with the acceleration factor determined using the rate-limiting step model (Eq. 7 and 8).

**Acceleration factors from experimental data:** The acceleration factors $a$ describe the ratio of the basal GTPase cycle rate (i.e. the rate of Cdc42 alone, $r_{c,0}$) over the GTPase cycle rate with one effector present (a GEF or a GAP) ($r_{c,1}$) (Eq. 6). We determined the rates using an exponential fit

$$[\text{GTP}]_t = [\text{GTP}]_{0h} \exp(-Kt)$$
$$\text{using } [GTP]_{0h} = 1$$
$$\text{and } K = K_1 + K_2 + K_{3,X} = k_1[\text{Cdc42}] + k_2[\text{Cdc42}])^2 + k_{3,X}[\text{Cdc42}][\text{X}]^m$$

where $K$ refers to the overall GTP hydrolysis rate and $X$ is the effector protein (i.e. Cdc24, Rga2$_{(I)}$, Rga2$_{(II)}$, or the GAP domain). Without effectors present ($[X] = 0$) $K = r_{c,0}$, and with an effector present $K = r_{c,1}$.
$K$ is concentration dependent and we here want to calculate it for maximal acceleration of the overall rate through GEF or GAP. Thus, we use 0.3 µM for Cdc24 (the highest Cdc24 concentration used in our assays) and 1.2 µM for all GAPs (given that the linear regime for both Rga2 versions extends up to 1.2 µM [2] ). Because the majority of assays were conducted using 1.3 µM Cdc42, we use 1.3 µM for Cdc42. Thus, the rates are:

- $K_1 + K_2 = k_1$ [Cdc42] + $k_2$ [Cdc42]$^2$ = 0.165 h$^{-1}$
- $K_{3,Cdc24} = k_{3,Cdc24}$ [Cdc42] [Cdc24]$^2$ = 1.391 h$^{-1}$
- $K_{3,Rga2-I} = k_{3,Rga2-I}$ [Cdc42] [Rga2$_{(I)}$] = 0.186 h$^{-1}$
- $K_{3,Rga2-II} = k_{3,Rga2-II}$ [Cdc42] [Rga2$_{(II)}$] = 0.434 h$^{-1}$
- $K_{3,GAPdomain} = r_{c,0} + k_{3,GAPdomain}$ [Cdc42] [GAP domain] = 0.282 h$^{-1}$

Using these rates, we can calculate the acceleration factors of GEF and GAP based on our experimental data (Eq. 6).

**Comparison of acceleration factors:** We now compare the experimentally determined acceleration factors of GEF and GAP (using protein concentrations leading to maximal accelerations of the overall rate) with the acceleration factors determined using the rate-limiting step model (Eq. 7 and 8).
Thus, we check if the acceleration by the GEF limits the maximum acceleration by the GAP in the way predicted by the limiting step model:

1. We calculate the acceleration of the GEF based on our experimental data: $a_{GEF} = \frac{r_{c,1}}{r_{c,0}} = \frac{K_1+K_2+K_{3,Cdc24}}{K_1+K_2} = 1 + \frac{K_{3,Cdc24}}{K_1+K_2}$.
2. We use Eq. 8 to calculate the maximum acceleration $a_{GAP}$ that the GAP could achieve in the rate-limiting step model.
3. We compare $a_{GAP}$ (rate-limiting step model) to $a_{GAP}$ calculated from experimental data (e.g. for Rga2$_{(I)}$: $a_{GAP} = \frac{r_{c,1}}{r_{c,0}} = \frac{K_1+K_2+K_{3,Rga2-I}}{K_1+K_2} = 1 + \frac{K_{3,Rga2-I}}{K_1+K_2}$). The rate-limiting step model describes the data fully if $a_{GAP}$ (rate-limiting step model) ≥ $a_{GAP}$ (experiment).

Analogously, we check if the acceleration by the GAP limits the maximum acceleration by the GEF in the way predicted by the rate-limiting step model:

1. We calculate the acceleration of the GAP based on our experimental data: e.g. for Rga2$_{(I)}$: $a_{GAP} = 1 + \frac{K_{3,Rga2-I}}{K_1+K_2}$.
2. We use Eq. 7 to calculate the maximum acceleration $a_{GEF}$ that the GEF could achieve in the rate-limiting step model.
3. We compare $a_{GEF}$ (rate-limiting step model) to $a_{GEF}$ calculated from experimental data: $a_{GEF} = 1 + \frac{K_{3,Cdc24}}{K_1+K_2}$. The rate-limiting step model describes the data fully if $a_{GEF}$ (rate-limiting step model) ≥ $a_{GEF}$ (experiment).

[2]We observed a linear regime for the GAP domain also above 1.2 µM. To keep the comparison for all GAPs consistent, we still use 1.2 µM for the GAP domain.

If one of the two aforementioned conditions on the rate-limiting effect of GAP/GEF addition is true, the rate-limiting step model describes our data fully and can be the sole source of the synergy term. The results are summarized in Appendix Tab. S5: We determined a 9.43-fold increase the total rate from the Cdc42 + GEF assay data (experiment, Cdc42 + GEF). A ~10-fold acceleration factor of the GEF would maximize the GAP acceleration factor to 1.12 (rate-limiting step model, Cdc42 + GAP). For GAPs, we observe acceleration factors of 2.13 (Rga2$_{(I)}$), 3.63 (Rga2$_{(II)}$), and 2.71 (GAP domain) (experiment, GAP), which are all significantly larger than the by the rate-limiting step model predicted acceleration factor (i.e. $a_{GAP}$ (rate-limiting step model) $\leq a_{GAP}$ (experiment)).
Similarly, the 2.13-fold (Rga2$_{(I)}$), 3.63-fold (Rga2$_{(II)}$), and 2.71-fold (GAP domain) increase the total rate from the Cdc42 + GAP assay data (experiment, Cdc42 + GAP) maximizes the GEF acceleration factor to 1.88 (Rga2$_{(I)}$), 1.38 (Rga2$_{(II)}$), and 1.58 (GAP domain) (rate-limiting step model, Cdc42 + GEF). For Cdc24, we observe an acceleration factor of 9.43 (experiment, Cdc42 + GEF). Here again, the experimentally observed rate acceleration by the GEF Cdc24 is much larger than the maximum acceleration predicted by the rate-limiting step model (i.e. $a_{GEF}$ (rate-limiting step model) $\leq a_{GEF}$ (experiment)).
Thus, our experimental data does not fit the interdependence of the acceleration achieved by the GEF or GAP in the way described by the rate-limiting step model - the synergy term cannot be fully explained by the rate-limiting step model alone. Furthermore, in Eq. 7 we assume that the GEF accelerates the cycle so much that the nucleotide exchange step is much faster than the hydrolysis step, at which point the effect of adding more GEF would saturate. We do not observe a GEF concentration regime where we see saturation. Thus in reality, the experimentally measured acceleration factor is likely an underestimation of the acceleration maximally possible, making the incompatibility between the rate-limiting step model and the acceleration factor data more pronounced.
Analogously, in Eq. 8 we assume that GAP accelerates the cycle so much that the hydrolysis step is much faster than the nucleotide exchange step, at which point the effect of adding more GAP would saturate. We do not observe a concentration regime for the GAP domain where we see saturation[3]. Thus, in reality the experimentally measured acceleration factor is likely an underestimation of the acceleration maximally possible.

[3]We observe saturation for Rga2, but believe that this saturation is due to auto-inhibition of Rga2. If this saturation where because the hydrolysis step cannot go faster/ is reaching a rate-limiting step, we would also observe saturation for the GAP domain (which we do not).

**Appendix Table S 5. The rate-limiting step model:** Comparison of acceleration factors determined experimentally (Eq. 6) with those following the rate-limiting step model (Eq. 7, 8).

| | **Cdc42 + GEF** | **Cdc42 + GAP** | | |
|---|---|---|---|---|
| | GEF = Cdc24 | GAP = Rga2$_{(I)}$ | GAP = Rga2$_{(II)}$ | GAP = GAP domain |
| **experiment:**<br>$a = \frac{r_{c,1}}{r_{c,0}} = 1 + \frac{K_{3,X}}{K_1+K_2}$ | $a_{GEF} = 1 + \frac{K_{3,Cdc24}}{K_1+K_2}$<br>= 9.43 | $a_{GAP} = 1 + \frac{K_{3,Rga2-I}}{K_1+K_2}$<br>= 2.13 | $a_{GAP} = 1 + \frac{K_{3,Rga2-II}}{K_1+K_2}$<br>= 3.63 | $a_{GAP} = 1 + \frac{K_{3,GAPdomain}}{K_1+K_2}$<br>= 2.71 |
| **rate-limiting step model:**<br>$a_{GEF} = \frac{a_{GAP}}{a_{GAP}-1}$<br>and<br>$a_{GAP} = \frac{a_{GEF}}{a_{GEF}-1}$ | GAP = Rga2$_{(I)}$: $a_{GEF}$ = 1.88<br>GAP = Rga2$_{(II)}$: $a_{GEF}$ = 1.38<br>GAP = GAP domain: $a_{GEF}$ = 1.58 | $a_{GAP}$ = 1.12 | | |

MGSSHHHHHHSSGLVPRGSHMASMTGGQQMGRGSEFDDDDKMSADPINDQ
SSLCVRCNKSIASSQVYELESKKWHDQCFTCYKCDKKLNADSDFLVLDIG
TLICYDCSDKCTNCGDKIDDTAIILPSSNEAYCSNCFRCCRCSNRIKNLK
YAKTKRGLCCMDCHEKLLRKKQLLLENQTKNSSKEDFPIKLPERSVKRPL
SPTRINGKSDVSTNNTAISKNLVSSNEDQQLTPQVLVSQERDESSLNDNN
DNDNSKDREETSSHARTVSIDDILNSTLEHDSNSIEEQSLVDNEDYINKM
GEDVTYRLLKPQRANRDSIVVKDPRIPNSNSNANRFFSIYDKEETDKDDT
DNKENEIIVNTPRNSTDKITSPLNSPMAVQMNEEVEPPHGLALTLSEATK
ENNKSSQGIQTSTSKSMNHVSPITRTDTVEMKTSTSSSTLRLSDNGSFSR
PQTADNLLPHKKVAPSPNKKLSRSFSLKSKNFVHNLKSKTSEMLDPKHPH

Fragment 1
= 49 kDa

HSTSIQESDTHSGWGVSSTHTNIRKSKAKKNPVSRGQSDSTIYNTLPQHG
NFTVPEFNHKKAQSSLGSISKKQNSNDTATNRRINGSFTSSSSGHHIAMF
RTPPLESGPLFKRPSLSSESAHHRSSSLQTSRSTNALLEDDSTKVDATDE
SATSLEKDFYFTELTLRKLKLDVRELEGTKKKLLQDVENLRLAKERLLND
VDNLTREKDKQSASSRESLEQKENIATSITVKSPSSNSDRKGSISNASPK

Fragment 2
= 26 kDa

PRFWKIFSSAKDHQVGDLESQQRSPNSSSGGTTNIAQKEISSPKLIRVHD
ELPSPGKVPLSPSPKRLDYTPDGSHLYGSSLQARCAYEKSTVPIIIRCCI
DRIEKDDIGLNMEGLYRKSGSQTLVEEIENEFAQNNSLHSDTLSPKLNAL
LNQDIHAVASVLKRYLRKLPDPVLSFSIYDALIDLVRNNQLIERLPLNND
KFLDSPQKVTIYEMVLKSLLEIFKILPVEHQEVLKVLAAHIGKVRRCSER
NLMNLHNLSLVFAPSLIHDFDGEKDIVDMKERNYIVEFILGNYRDIFKQA

Fragment 3
= 33 kDa

**Appendix Figure S 9. Amino acid sequence of the Rga2$_{(I)}$ construct.** Protein fragments that are not be visible on stain-free visualized SDS-PAGE (detection of tryptophan) or in anti-His Western blots are annotated on the right. The following sequence features are highlighted: 6His tag (blue), tryptophan (W) (red with yellow background), LIM zinc-binding domain (underlined, wriggled line), LIM zinc-binding domain (underlined, dotted line), RhoGAP domain (according to UNIPROT: underlined, single line; according to Smith *et al.* [Smith et al., 2002]: underlined, double line).

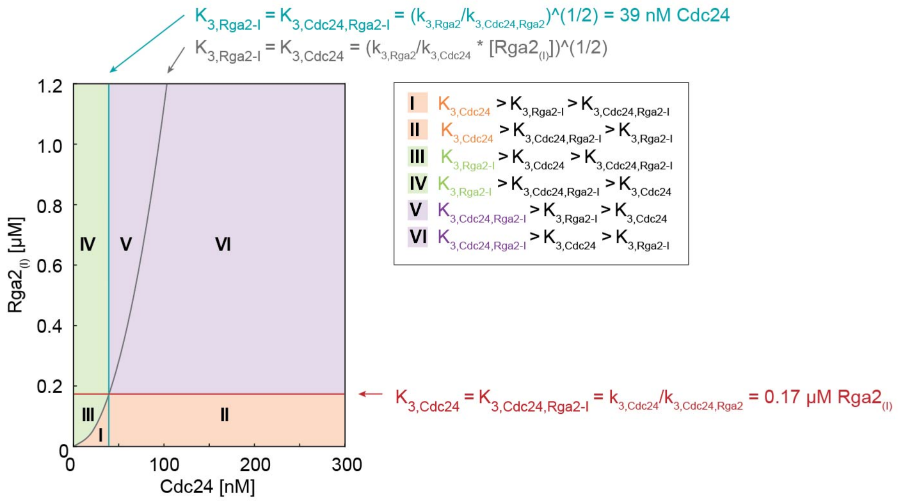


**Appendix Figure S 10. Regime diagram illustrating the dominant rate $K$ across varying Cdc24 and Rga2$_{(I)}$ concentrations.** The diagram was generated using rates $k_3$ for effectors Cdc24 and Rga2$_{(I)}$ given in Tab. 3.

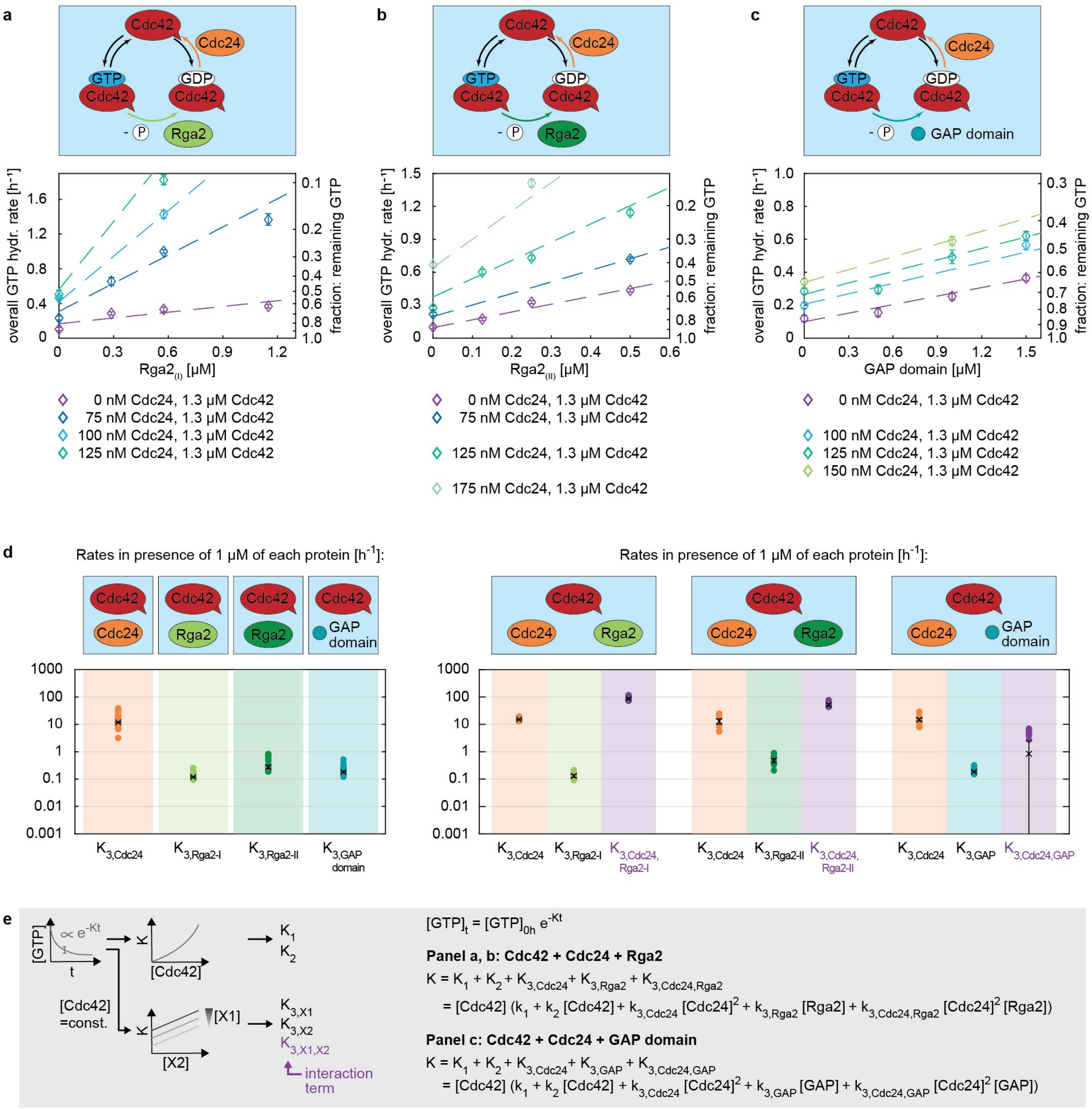


**Appendix Figure S 11. The C-terminal Flag-tag of Rga2$_{(II)}$ might weaken Rga2-Cdc24 binding, reducing Cdc24-Rga2$_{(II)}$ synergy.** (a-c) GTPase assay data of (a) Cdc42-Cdc24-Rga2$_{(I)}$, (b) Cdc42-Cdc24-Rga2$_{(II)}$, and (c) Cdc42-Cdc24-GAP domain mixtures. (d) Summary of the rates $K$ obtained in the three-protein assay (right) in comparison to those of the two-protein assay (left): In three-protein assays the rate contribution of the individual proteins is comparable to those obtained in the two-protein assay. Additionally, an interaction rate is obtained (shown in purple). Both Cdc42-Cdc24-Rga2$_{(I)}$ and Cdc42-Cdc24-Rga2$_{(I)}$ show a positive interaction rate, with $K_{3,Cdc24,Rga2-I} > K_{3,Cdc24,Rga2-II}$ (Tab. 3). The C-terminal Flag-tag of Rga2$_{(II)}$ might weaken Cdc24-Rga2 binding, reducing Cdc24-Rga2$_{(II)}$ synergy. The values shown refer to the rate values in presence of 1 μM of each protein, e.g. '$K_{3,Cdc24}$' refers to '$k_{3,Cdc24}$ [Cdc24]$^2$ [Cdc42]' with [Cdc42]=[Cdc24]=1 μM. Crosses with error bars represent the weighted mean and the standard error of the mean (Appendix Supplementary Text 1), and filled circles show individual measurements. (e) Illustration of the data processing and fitting model.

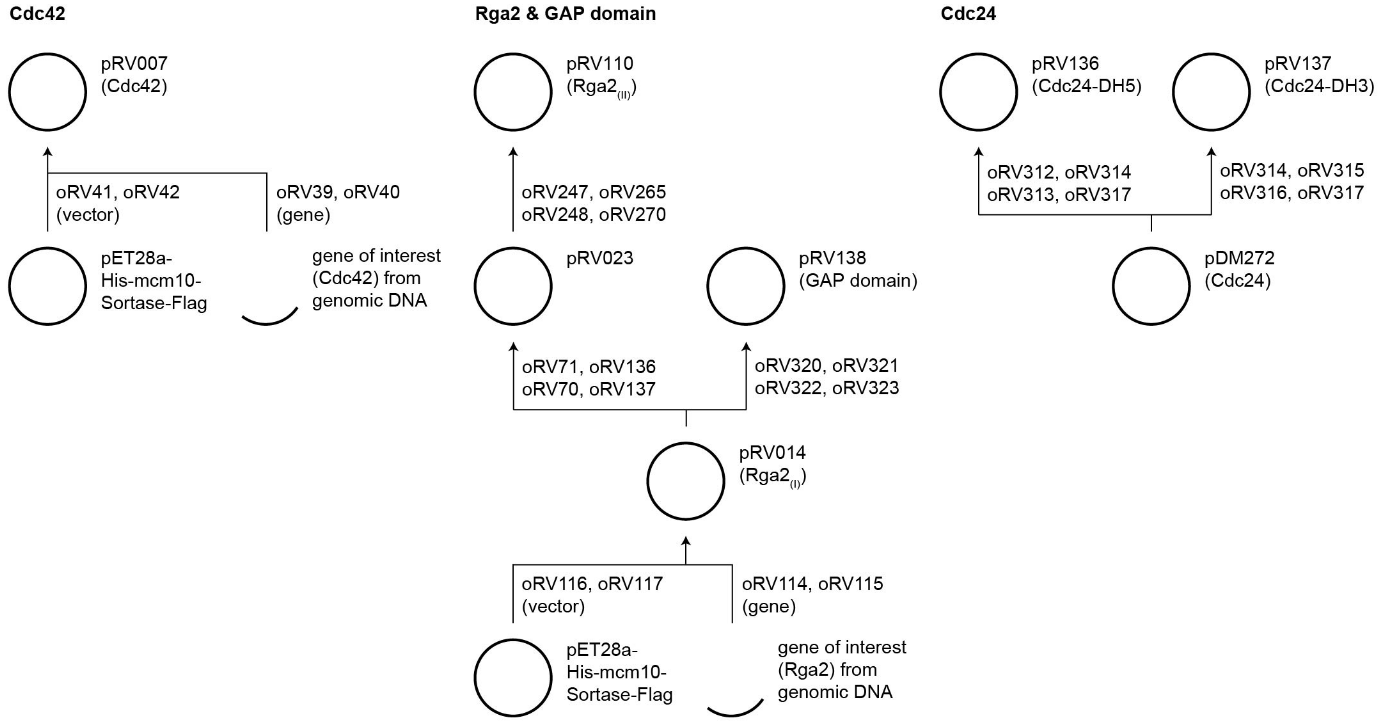


**Appendix Figure S 12. Schematic of the plasmid construction.** pET28a-His-mcm10-Sortase-Flag was received from N. Dekker (TU Delft) and is based on pBP6 [Douglas and Diffley, 2016]. pDM272 was received from D. McCusker (University of Bordeaux) [Rapali et al., 2017].

**Appendix Table S 6. Primer overview.**

| Primer | Sequence |
|---|---|
| oRV39 | Gcaaatgggt cgcggatccg aattcGATGA CGACGATAAA ATGCAAACGC TAAAGTGTGT TGTTG |
| oRV40 | CACCGTCGTG GTCCTTGTAG TCACCGCCGG TTTCCGGTAA CAAAATTGCA CATTTTTTAC TTTTCTTGAT AACAGG |
| oRV41 | TTACCGGAAA CCGGCGGT |
| oRV42 | TTTATCGTCG TCATCgaatt cggatcc |
| oRV70 | caacgcggcc tttttacggt tcc |
| oRV71 | ggaaccgtaa aaaggccgcg ttg |
| oRV114 | cgcggatccg aattcGATGA CGACGATAAA ATGTCAGCTG ACCCTATTAA TGACCAATCG TCTTTATG |
| oRV115 | GTTTCCGGTA AGCTTCCTCC GCCACCTTAT GCTTGCTTAA ATATGTCTCT ATAGTTTCCA A |
| oRV116 | TTGGAAACTA TAGAGACATA TTTAAGCAAG CATAAGGTGG CGGAGGAAGC TTACCGGAAA C |
| oRV117 | CATAAAGACG ATTGGTCATT AATAGGGTCA GCTGACATTT TATCGTCGTC ATCgaattcg gatccgcg |
| oRV136 | TTGGAAACTA TAGAGACATA TTTAAGCAAG CAGGTGGCGG AGGAAGCTTA CCGGAAAC |
| oRV137 | GTTTCCGGTA AGCTTCCTCC GCCACCTGCT TGCTTAAATA TGTCTCTATA GTTTCCAA |
| oRV247 | ggcgcagcgg tcgggctgaa cggggggtt |
| oRV248 | aacccccgt tcagcccgac cgctgcgcc |
| oRV265 | accttgaaaa tagagattct cgaattcgga tccgcgaccc atttgCtgtc cacc |
| oRV270 | gagaatctct attttcaagg tATGTCAGCT GACCCTATTA ATGACCAATC GTCTTTATGT G |
| oRV312 | ggaaggttct actaaagcat ttatCGCCGC ggatattaga aatcttcttt gaaaatc |
| oRV313 | gaagatttct aatatccGCG GCGataaatg ctttagtaga accttcc |
| oRV314 | gggggttcgt gcacacagc |
| oRV315 | tcacccaaat taggCGCcaa catgtacaac tcttcagacg ttattag |
| oRV316 | cgtctgaaga gttgtacatg ttgGCGccta atttgggtga tgctatag |
| oRV317 | gctgtgtgca cgaaccccc |
| oRV320 | atgggcagca gccatcatca tcatcatcac agcagcggcG AAAAAAGTAC TGTTCCAATA ATTATCAGGT GCTGC |
| oRV321 | gcttgtcgac ggagctcgaa ttcTTAGCCG TCAAAATCGT GGATAAGACT CGG |
| oRV322 | CCGAGTCTTA TCCACGATTT TGACGGCTAA gaattcgagc tccgtcgaca agc |
| oRV323 | GCAGCACCTG ATAATTATTG GAACAGTACT TTTTTCgccg ctgctgtgat gatgatgatg atggctgctg cccat |

## Appendix Supplementary Text 5: Amino acid sequences of used proteins

Cdc42, pRV007:

MGSSHHHHHHSSGLVPRGSHMASMTGGQQMGRGSEFDDDDKMQTLKCVVV GDGAVGKTCLLISYTTNQFPADYVPTVFDNYAVTVMIGDEPYTLGLFDTA
GQEDYDRLRPLSYPSTDVFLVCFSVISPPSFENVKEKWFPEVHHHCPGVP CLVVGTQIDLRDDKVIIEKLQRQRLRPITSEQGSRLARELKAVKYVECSA
LTQRGLKNVFDEAIVAALEPPVIKKSKKCAILLPETGGDYKDHDGDYKDH DIDYKDDDDK

Rga2$_{(I)}$, pRV014:

MGSSHHHHHHSSGLVPRGSHMASMTGGQQMGRGSEFDDDDKMSADPINDQ SSLCVRCNKSIASSQVYELESKKWHDQCFTCYKCDKKLNADSDFLVLDIG
TLICYDCSDKCTNCGDKIDDTAIILPSSNEAYCSNCFRCCRCSNRIKNLK YAKTKRGLCCMDCHEKLLRKKQLLLENQTKNSSKEDFPIKLPERSVKRPL
SPTRINGKSDVSTNNTAISKNLVSSNEDQQLTPQVLVSQERDESSLNDNN DNDNSKDREETSSHARTVSIDDILNSTLEHDSNSIEEQSLVDNEDYINKM
GEDVTYRLLKPQRANRDSIVVKDPRIPNSNSNANRFFSIYDKEETDKDDT DNKENEIIVNTPRNSTDKITSPLNSPMAVQMNEEVEPPHGLALTLSEATK
ENNKSSQGIQTSTSKSMNHVSPITRTDTVEMKTSTSSSTLRLSDNGSFSR PQTADNLLPHKKVAPSPNKKLSRSFSLKSKNFVHNLKSKTSEMLDPKHPH
HSTSIQESDTHSGWGVSSTHTNIRKSKAKKNPVSRGQSDSTIYNTLPQHG NFTVPEFNHKKAQSSLGSISKKQNSNDTATNRRINGSFTSSSSGHHIAMF
RTPPLESGPLFKRPSLSSESAHHRSSSLQTSRSTNALLEDDSTKVDATDE SATSLEKDFYFTELTLRKLKLDVRELEGTKKKLLQDVENLRLAKERLLND
VDNLTREKDKQSASSRESLEQKENIATSITVKSPSSNSDRKGSISNASPK PRFWKIFSSAKDHQVGDLESQQRSPNSSSGGTTNIAQKEISSPKLIRVHD
ELPSPGKVPLSPSPKRLDYTPDGSHLYGSSLQARCAYEKSTVPIIIRCCI DRIEKDDIGLNMEGLYRKSGSQTLVEEIENEFAQNNSLHSDTLSPKLNAL
LNQDIHAVASVLKRYLRKLPDPVLSFSIYDALIDLVRNNQLIERLPLNND KFLDSPQKVTIYEMVLKSLLEIFKILPVEHQEVLKVLAAHIGKVRRCSER
NLMNLHNLSLVFAPSLIHDFDGEKDIVDMKERNYIVEFILGNYRDIFKQA

Rga2$_{(II)}$, pRV110:

MGSSHHHHHHSSGLVPRGSHMASMTGGQQMGRGSEFENLYFQGMSADPIN DQSSLCVRCNKSIASSQVYELESKKWHDQCFTCYKCDKKLNADSDFLVLD
IGTLICYDCSDKCTNCGDKIDDTAIILPSSNEAYCSNCFRCCRCSNRIKN LKYAKTKRGLCCMDCHEKLLRKKQLLLENQTKNSSKEDFPIKLPERSVKR
PLSPTRINGKSDVSTNNTAISKNLVSSNEDQQLTPQVLVSQERDESSLND NNDNDNSKDREETSSHARTVSIDDILNSTLEHDSNSIEEQSLVDNEDYIN
KMGEDVTYRLLKPQRANRDSIVVKDPRIPNSNSNANRFFSIYDKEETDKD DTDNKENEIIVNTPRNSTDKITSPLNSPMAVQMNEEVEPPHGLALTLSEA
TKENNKSSQGIQTSTSKSMNHVSPITRTDTVEMKTSTSSSTLRLSDNGSF SRPQTADNLLPHKKVAPSPNKKLSRSFSLKSKNFVHNLKSKTSEMLDPKH
PHHSTSIQESDTHSGWGVSSTHTNIRKSKAKKNPVSRGQSDSTIYNTLPQ HGNFTVPEFNHKKAQSSLGSISKKQNSNDTATNRRINGSFTSSSSGHHIA
MFRTPPLESGPLFKRPSLSSESAHHRSSSLQTSRSTNALLEDDSTKVDAT DESATSLEKDFYFTELTLRKLKLDVRELEGTKKKLLQDVENLRLAKERLL
NDVDNLTREKDKQSASSRESLEQKENIATSITVKSPSSNSDRKGSISNAS PKPRFWKIFSSAKDHQVGDLESQQRSPNSSSGGTTNIAQKEISSPKLIRV
HDELPSPGKVPLSPSPKRLDYTPDGSHLYGSSLQARCAYEKSTVPIIIRC CIDRIEKDDIGLNMEGLYRKSGSQTLVEEIENEFAQNNSLHSDTLSPKLN
ALLNQDIHAVASVLKRYLRKLPDPVLSFSIYDALIDLVRNNQLIERLPLN NDKFLDSPQKVTIYEMVLKSLLEIFKILPVEHQEVLKVLAAHIGKVRRCS
ERNLMNLHNLSLVFAPSLIHDFDGEKDIVDMKERNYIVEFILGNYRDIFK QAGGGGSLPETGGDYKDHDGDYKDHDIDYKDDDDK

GAP domain (amino acids 797-981 from Rga2 [Smith et al., 2002]), pRV138:

MGSSHHHHHHSSGEKSTVPIIIRCCIDRIEKDDIGLNMEGLYRKSGSQTL VEEIENEFAQNNSLHSDTLSPKLNALLNQDIHAVASVLKRYLRKLPDPVL
SFSIYDALIDLVRNNQLIERLPLNNDKFLDSPQKVTIYEMVLKSLLEIFK ILPVEHQEVLKVLAAHIGKVRRCSERNLMNLHNLSLVFAPSLIHDFDG

Cdc24, pDM272:

MAIQTRFASGTSLSDLKPKPSATSISIPMQNVMNKPVTEQDSLFHICANI RKRLEVLPQLKPFLQLAYQSSEVLSERQSLLLSQKQHQELLKSNGANRDS
SDLAPTLRSSSISTATSLMSMEGISYTNSNPSATPNMEDTLLTFSMGILP ITMDCDPVTQLSQLFQQGAPLCILFNSVKPQFKLPVIASDDLKVCKKSIY
DFILGCKKHFAFNDEELFTISDVFANSTSQLVKVLEVVETLMNSSPTIFP SKSKTQQIMNAENQHRHQPQQSSKKHNEYVKIIKEFVATERKYVHDLEIL
DKYRQQLLDSNLITSEELYMLFPNLGDAIDFQRRFLISLEINALVEPSKQ RIGALFMHSKHFFKLYEPWSIGQNAAIEFLSSTLHKMRVDESQRFIINNK
LELQSFLYKPVQRLCRYPLLVKELLAESSDDNNTKELEAALDISKNIARS INENQRRTENHQVVKKLYGRVVNWKGYRISKFGELLYFDKVFISTTNSSS
EPEREFEVYLFEKIIILFSEVVTKKSASSLILKKKSSTSASISASNITDN NGSPHHSYHKRHSNSSSSNNIHLSSSSAAAIIHSSTNSSDNNSNNSSSSS
LFKLSANEPKLDLRGRIMIMNLNQIIPQNNRSLNITWESIKEQGNFLLKF KNEETRDNWSSCLQQLIHDLKNEQFKARHHSSTSTTSSTAKSSSMMSPTT
TMNTPNHHNSRQTHDSMASFSSSHMKRVSDVLPKRRTTSSSFESEIKSIS ENFKNSIPESSILFRISYNNNSNNTSSSEIFTLLVEKVWNFDDLIMAINS
KISNTHNNNISPITKIKYQDEDGDFVVLGSDEDWNVAKEMLAENNEKFLN IRLYLEHHHHHH

Cdc24-DH5 (Cdc24 mutation F322A [Mionnet et al., 2008]), pRV136:

MAIQTRFASGTSLSDLKPKPSATSISIPMQNVMNKPVTEQDSLFHICANI RKRLEVLPQLKPFLQLAYQSSEVLSERQSLLLSQKQHQELLKSNGANRDS
SDLAPTLRSSSISTATSLMSMEGISYTNSNPSATPNMEDTLLTFSMGILP ITMDCDPVTQLSQLFQQGAPLCILFNSVKPQFKLPVIASDDLKVCKKSIY
DFILGCKKHFAFNDEELFTISDVFANSTSQLVKVLEVVETLMNSSPTIFP SKSKTQQIMNAENQHRHQPQQSSKKHNEYVKIIKEFVATERKYVHDLEIL
DKYRQQLLDSNLITSEELYMLFPNLGDAIDFQRRFLISAAINALVEPSKQ RIGALFMHSKHFFKLYEPWSIGQNAAIEFLSSTLHKMRVDESQRFIINNK
LELQSFLYKPVQRLCRYPLLVKELLAESSDDNNTKELEAALDISKNIARS INENQRRTENHQVVKKLYGRVVNWKGYRISKFGELLYFDKVFISTTNSSS
EPEREFEVYLFEKIIILFSEVVTKKSASSLILKKKSSTSASISASNITDN NGSPHHSYHKRHSNSSSSNNIHLSSSSAAAIIHSSTNSSDNNSNNSSSSS
LFKLSANEPKLDLRGRIMIMNLNQIIPQNNRSLNITWESIKEQGNFLLKF KNEETRDNWSSCLQQLIHDLKNEQFKARHHSSTSTTSSTAKSSSMMSPTT
TMNTPNHHNSRQTHDSMASFSSSHMKRVSDVLPKRRTTSSSFESEIKSIS ENFKNSIPESSILFRISYNNNSNNTSSSEIFTLLVEKVWNFDDLIMAINS
KISNTHNNNISPITKIKYQDEDGDFVVLGSDEDWNVAKEMLAENNEKFLN IRLYLEHHHHHH

Cdc24-DH3 (Cdc24 mutations L339A and E340A [Mionnet et al., 2008]), pRV137:

MAIQTRFASGTSLSDLKPKPSATSISIPMQNVMNKPVTEQDSLFHICANI RKRLEVLPQLKPFLQLAYQSSEVLSERQSLLLSQKQHQELLKSNGANRDS
SDLAPTLRSSSISTATSLMSMEGISYTNSNPSATPNMEDTLLTFSMGILP ITMDCDPVTQLSQLFQQGAPLCILFNSVKPQFKLPVIASDDLKVCKKSIY
DFILGCKKHFAFNDEELFTISDVFANSTSQLVKVLEVVETLMNSSPTIFP SKSKTQQIMNAENQHRHQPQQSSKKHNEYVKIIKEFVATERKYVHDLEIL
DKYRQQLLDSNLITSEELYMLAPNLGDAIDFQRRFLISLEINALVEPSKQ RIGALFMHSKHFFKLYEPWSIGQNAAIEFLSSTLHKMRVDESQRFIINNK
LELQSFLYKPVQRLCRYPLLVKELLAESSDDNNTKELEAALDISKNIARS INENQRRTENHQVVKKLYGRVVNWKGYRISKFGELLYFDKVFISTTNSSS
EPEREFEVYLFEKIIILFSEVVTKKSASSLILKKKSSTSASISASNITDN NGSPHHSYHKRHSNSSSSNNIHLSSSSAAAIIHSSTNSSDNNSNNSSSSS
LFKLSANEPKLDLRGRIMIMNLNQIIPQNNRSLNITWESIKEQGNFLLKF KNEETRDNWSSCLQQLIHDLKNEQFKARHHSSTSTTSSTAKSSSMMSPTT
TMNTPNHHNSRQTHDSMASFSSSHMKRVSDVLPKRRTTSSSFESEIKSIS ENFKNSIPESSILFRISYNNNSNNTSSSEIFTLLVEKVWNFDDLIMAINS
KISNTHNNNISPITKIKYQDEDGDFVVLGSDEDWNVAKEMLAENNEKFLN IRLYLEHHHHHH

The protein constructs contain some of the following features:

- 6His-tag: HHHHHH
- Flag-tag: DYKDHDGDYKDHDIDYKDDDDK
- Thrombin cut site: LVPRGS
- Enterokinase cut site: DDDDK
- TEV cut site: ENLYFQG
- Sortase cut/ligation site: LPETGG
- T7 tag (to aid protein expression): MASMTGGQQMGRGSEF

More information on the protein constructs is given in [preprint: Tschirpke et al., 2023].

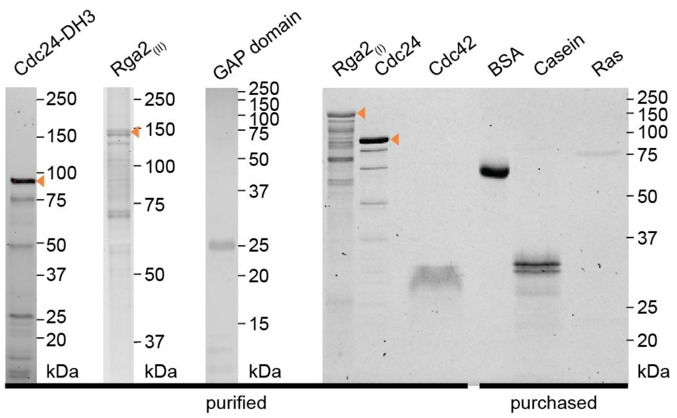


**Appendix Figure S 13. SDS-PAGE of used proteins.** An orange arrow indicates the band of the correct size. The GAP domain is visualized using SimplyBlue SafeStain while all other gels show data obtained from stain-free imaging.

## References

Chebotareva, N. A., Kurganov, B. I., and Livanova, N. B. (2004). Biochemical Effects of Molecular Crowding. *Biochemistry (Moscow)*, 69(11):1239–1251.

Chiou, J.-g., Balasubramanian, M. K., and Lew, D. J. (2017). Cell Polarity in Yeast. *Annual Review of Cell and Developmental Biology*, 33:77–101.

Douglas, M. E. and Diffley, J. F. (2016). Recruitment of Mcm10 to sites of replication initiation requires direct binding to the minichromosome maintenance (MCM) complex. *Journal of Biological Chemistry*, 291(11):5879–5888.

Heij, C., de Boer, P., Franses, P. H., Kloek, T., and van Dijk, H. K. (2004). *Econometric Methods with Applications in Business and Economics*. Oxford University Press.

Kang, P. J., Béven, L., Hariharan, S., and Park, H. O. (2010). The Rsr1/Bud1 GTPase interacts with itself and the Cdc42 GTPase during bud-site selection and polarity establishment in budding yeast. *Molecular Biology of the Cell*, 21(17):3007–3016.

Martin, S. G. (2015). Spontaneous cell polarization: Feedback control of Cdc42 GTPase breaks cellular symmetry. *BioEssays*, 37(11):1193–1201.

Mionnet, C., Bogliolo, S., and Arkowitz, R. A. (2008). Oligomerization regulates the localization of Cdc24, the Cdc42 activator in Saccharomyces cerevisiae. *Journal of Biological Chemistry*, 283(25):17515–17530.

Park, H.-O. and Bi, E. (2007). Central Roles of Small GTPases in the Development of Cell Polarity in Yeast and Beyond. *Microbiology and Molecular Biology Reviews*, 71(1):48–96.

Rapali, P., Mitteau, R., Braun, C., Massoni-Laporte, A., Ünlü, C., Bataille, L., Arramon, F. S., Gygi, S. P., and McCusker, D. (2017). Scaffold-mediated gating of Cdc42 signalling fl ux. *eLife*, 6:1–18.

Shimada, Y., Wiget, P., Gulli, M. P., Bi, E., and Peter, M. (2004). The nucleotide exchange factor Cdc24p may be regulated by auto-inhibition. *EMBO Journal*, 23(5):1051–1062.

Smith, G. R., Givan, S. A., Cullen, P., and Sprague, G. F. (2002). GTPase-Activating Proteins for Cdc42. *Eukaryotic Cell*, 1(3):469–480.

Tschirpke, S., Daalman, W. K., and Laan, L. (2024). Quantification of GTPase Cycling Rates of GTPases and GTPase:Effector Mixtures Using GTPase Glo A ssays. *Current Protocols*, 4(4):1–29.

Tschirpke, S., van Opstal, F., van der Valk, R., Daalman, W. K.-G., and Laan, L. (2023). A guide to the in vitro reconstitution of Cdc42 activity and its regulation. *BioRxiv* doi: 10.1101/2023.04.24.538075 [PREPRINT].

Zhang, B., Gao, Y., Moon, S. Y., Zhang, Y., and Zheng, Y. (2001). Oligomerization of Rac1 GTPase Mediated by the Carboxyl-terminal Polybasic Domain. *Journal of Biological Chemistry*, 276(12):8958–8967.

Zhang, B., Zhang, Y., Collins, C. C., J ohnson, D. I., and Zheng, Y. (1999). A built-in arginine finger triggers the self-stimulatory GTPase- activating activity of Rho family GTPases. *Journal of Biological Chemistry*, 274(5):2609–2612.

Zhang, B. and Zheng, Y. (1998). Negative regulation of Rho family GTPases Cdc42 and Rac2 by homodimer formation. *Journal of Biological Chemistry*, 273(40):25728–25733.
