# Supplementary figures and images for: Oligomerization-dependent and synergistic regulation of Cdc42 GTPase cycling by a GEF and a GAP
Source: EMBO Rep. 2026 Feb 9;27(6):1463–77. doi: 10.1038/s44319-026-00695-7 (PMC13022455; doi:10.1038/s44319-026-00695-7)

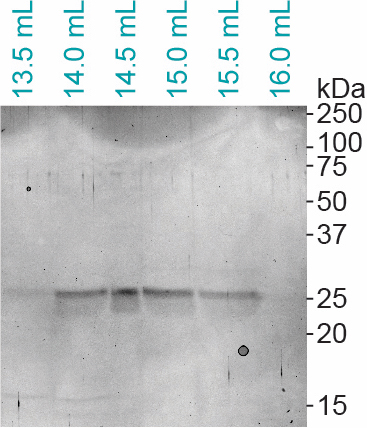

Supplement: Supplementary file 4 — Source data Fig. 4 [file 44319_2026_695_MOESM4_ESM.zip › Figure 4/4A/GAP domain Silver stain.jpg]

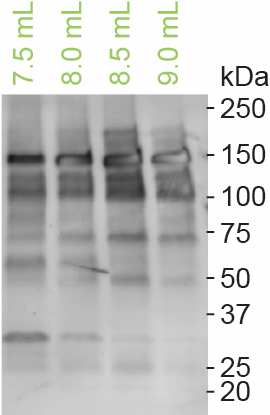

Supplement: Supplementary file 4 — Source data Fig. 4 [file 44319_2026_695_MOESM4_ESM.zip › Figure 4/4A/Rga2_I anti-His blot.jpg]

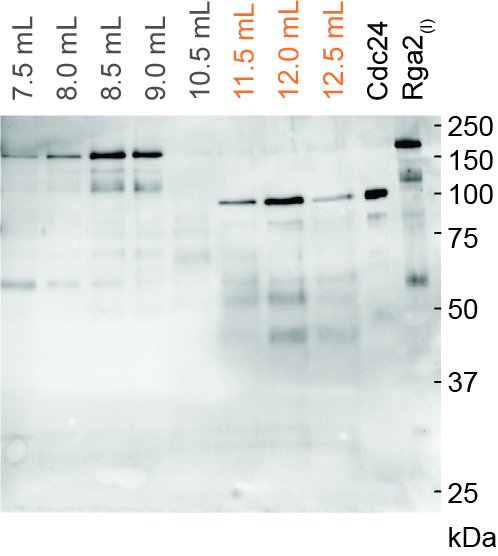

Supplement: Supplementary file 6 — Source data Fig. 7 [file 44319_2026_695_MOESM6_ESM.zip › Figure 7/7B/anti-His blot.jpg]

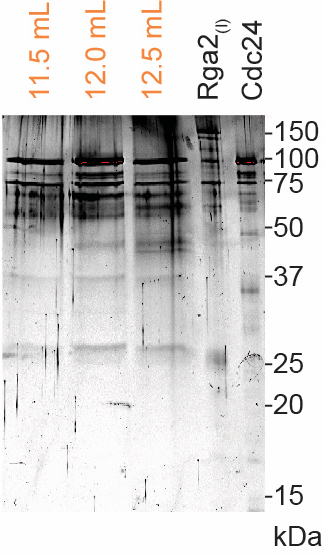

Supplement: Supplementary file 6 — Source data Fig. 7 [file 44319_2026_695_MOESM6_ESM.zip › Figure 7/7B/SYPRO Ruby.jpg]
